# Personalized 3D surgical planning in liver transplantation: A new era in preoperative assessment and management of vascular and biliary complications

**DOI:** 10.1007/s00423-026-03984-w

**Published:** 2026-02-27

**Authors:** Victor Lopez-Lopez, Cecilia Maina, Lucía Hernández-Ramos, Pedro Cascales-Campos, Alberto Baroja-Mazo, Dilmurodjon Eshmuminov, Jose Antonio-Pons, Guillermo Carbonell, Sergio Hernández-Kakauridze, Miguel Rodriguez-Velázquez, Francisco Sánchez-Bueno, Ricardo Robles-Campos, Miguel A. Gómez-Bravo, Pablo Ramírez

**Affiliations:** 1 Department of General and Digestive Surgery, IMIB-Pascual Parrilla, Virgen de la Arrixaca University Hospital, Murcia, Spain; 2 Digestive and Endocrine Surgery and Transplantation of Abdominal Organs Research Group, Biomedical Research Institute of Murcia (IMIB), Murcia, Spain; 3 Department of Surgery and Transplantation, University Hospital Zurich and University of Zurich, Zurich, Switzerland; 4 Department of Hepatology, IMIB- Pascual Parrilla, Virgen de la Arrixaca University Hospital, Murcia, Spain; 5 Department of Radiology, IMIB- Pascual Parrilla, Virgen de la Arrixaca University Hospital, Murcia, Spain; 6 Innovation and Technology Department, Cella Medical Solutions, Murcia, Spain; 7 Catholic University of Murcia (UCAM), Murcia, Spain; 8 Hepatobiliary Unit of Surgery and Liver Transplatation, Virgen del Rocío Hospital, Sevilla, Spain; 9 Department of Hepatobiliary Surgery, San Raffaele Hospital, Milan, Italy

**Keywords:** Liver transplantation, Vascular complications, Biliary complications, Decision-making, 3D model

## Abstract

**Purpose:**

This study aimed to analyze next-generation 3D modeling software utility for personalized surgery in the field of liver transplantation.

**Methods:**

A retrospective cross-sectional pilot study was conducted at two referral centers including cases of liver transplantation-related complications – either preoperative portal vein thrombosis (PVT) or postoperative portal, arterial or biliary strictures – for which high-quality radiologic imaging was available. 3D model diagnostic accuracy was evaluated on arterial complications comparing the thrombosis/stenosis extension described by the virtual model and the actual site of the new arterial anastomosis at retransplantation (gold standard). In addition, the perceived benefit of 3D reconstructions was assessed through a questionnaire submitted to the experienced surgeons of the team (Likert scale, 1 to 5).

**Results:**

Thirty-nine patients were included in the study population: 17 with arterial thrombosis/stenosis, 11 with portal vein thrombosis and 11 with biliary strictures diagnoses. The concordance rate between 3D models’ virtual reproduction and intraoperative findings evaluated for arterial complications was 88.2%. According to the most experienced surgeons of the team, 3D models provide a high benefit in surgical planning (4.6/5 on a Likert scale).

**Conclusions:**

Virtual reconstructions are particularly useful in transplantation-related complication management, giving reliable representation of structures’ caliber, trajectory, and spatial relationships; identifying anatomical variants and locating the level, length, and severity of vascular and biliary strictures. In such a complex scenario, the 3D models’ visual clarity helps harmonize perspectives within the multidisciplinary team, fostering more informed and shared clinical decisions.

**Supplementary Information:**

The online version contains supplementary material available at 10.1007/s00423-026-03984-w.

## Introduction

Traditionally, surgical planning relies on established methods such as computed tomography (CT) and magnetic resonance imaging (MRI). Nevertheless, especially in complex procedures, there is a growing recognition of the potential benefits of augmenting these approaches using three-dimensional (3D) visualization techniques [1, 2]. The application of 3D imaging in guiding hepatobiliary and other abdominal surgeries has gained increasing significance; however, the experience in perioperative simulations in liver transplantation (LT) procedures is very scarce [3, 4]. Challenges include possible spleno-porto-mesenteric axis thrombosis, arterial variants, porto-systemic shunt, biliary or arterial complications, and retransplantation or preoperative planning of living liver donation. Spleno-porto-mesenteric axis thrombosis is found in 2% to 23% of cirrhotic patients [5] and is generally characterized by the thrombotic obstruction of portal vein (portal vein thrombosis – PVT), leading to vessel occlusion [6–8]. Reduced portal flow velocity and increased flow volume are independent risk factors for PVT development [9]. The severity of PVT is classified using the Yerdel grading system, with grades 1 to 3 manageable through transplant yielding outcomes similar to those without PVT; while grade 4 (< 3% of cases) may require technical modifications and advanced surgical resources [10, 11]. Vascular complications following LT, while infrequent, represent some of the most critical issues, often resulting in high rates of graft loss and even mortality [12]. Hepatic artery thrombosis (HAT) stands out as the most prevalent and severe arterial complication in LT (0–12%), accounting for approximately half of all arterial complications [12]. and standing as the primary cause of primary liver graft non-function [12]. In cases where revascularization is not performed, the retransplantation rate varies from 25% to 83%, carrying a mortality rate of up to 50% [12]. Biliary complications represent a substantial source of morbidity and mortality following LT [13, 14], reaching an incidence of 10–15% in the context of deceased donor transplantation. Surgical planning models are anatomical replicas generated from the imaging tests of each patient and employed by surgeons as preoperative tools, facilitating a comprehensive understanding of intricate anatomical structures. These models assist in delineating the precise surgical approach and contribute to optimizing surgical proficiency. The aim of this study was to analyze the utility of next-generation 3D modeling software for personalized surgery in the field of LT.

## Methods

### Study design

A retrospective cross-sectional pilot study was conducted to evaluate 3D modeling utility in the surgical planning of LT-related complications. All patients with LT-related complications recorded at Virgen de la Arrixaca University Hospital (Murcia, Spain) and Virgen del Rocio University Hospital (Seville, Spain) between 2002 and 2022, were evaluated. The inclusion criteria were: LT-related complication diagnosis – either preoperative portal vein thrombosis (PVT) or postoperative portal, arterial or biliary strictures – and availability of good quality radiological imaging. Patients were excluded if radiological imaging had not been performed or if the acquired images did not meet the standardized image acquisition protocols, preventing accurate reconstruction and analysis. Vascular complications were evaluated using at least a three-phase contrast-enhanced CT scan, although angiography was often employed in HAT cases. Biliary complications were investigated with MRI incorporating cholangiographic sequences. A 3D model was constructed retrospectively, based on the radiological studies available. The Institutional Review Board of the Clinic and University Hospital Virgen de la Arrixaca (Murcia, Spain) approved this study (Internal Protocol Code: (Internal Protocol Code: 2025-2-3-HCUVA). To analyze the utility of next-generation 3D modeling software, the degree of accordance between 3D models arterial reconstructions – interpreted by an expert radiologist – and intraoperative findings, namely the new anastomosis site, was evaluated. Moreover, surgeons perceived benefit brought by virtual reconstructions was assessed through a questionnaire based on a Likert scale (1 to 5).

### Imaging acquisition and processing

A 3D reconstruction of the images was created for each patient using Cella Medical Solutions service (3D-MSP^®^). Data were obtained in DICOM format from preoperative CT and MRI studies. CT images were acquired with intravenous contrast during the portal and arterial phases using a high-resolution multiplanar scan with a 512 × 512 matrix and a slice thickness of 0.5 mm. MRI, though not mandatory, was performed using a 1.5-T scanner with a 512 × 512 matrix, obtaining coronal and axial sections with the following liver-specific sequences: Fast Spin Echo T2 with fat suppression, T1 in-phase and out-of-phase, T2* GRE, diffusion-weighted imaging, and magnetic resonance cholangiopancreatography. Image fusion techniques were used to correct for patient breathing, motion, or positioning artifacts. A specialized team of engineers and radiologist processed the two-dimensional images using advanced, validated medical imaging algorithms (*Simpleware ScanIP software*). An AI model, based on convolutional neural networks, created a draft of the segmentation, that was then validated by technicians and radiologists specialized in 3D reconstructions. The resulting models were exported as stereolithographic files for further analysis, and post-processing of virtual reconstruction was performed.

### Virtual model development

The anatomical segmentations of the virtual model included an accurate representation of the hepatic parenchyma, hepatic arteries, portal and systemic venous vasculature, and biliary ducts. Furthermore, the lymph nodes, duodenum, and diaphragmatic pillars were incorporated into the model, providing a comprehensive and detailed illustration of the patient’s anatomy for precise surgical planning. Pathological structures were also included in the model, allowing exact visualization of thrombosis, portosystemic shunts, stenosis, cavernomatosis, atheromatous plaques, veins, arteries and shunt gauges. The model was analyzed using the surgical planner developed by Cella Medical Solutions©, integrating advanced tools specifically designed for hepatic surgery and transplantation. This software enables precise measurements of caliber and distance, which are crucial for evaluating the vascular and biliary structures. It also provides dynamic navigation through axial, coronal, and sagittal planes, allowing detailed and personalized visualization of patient anatomy. In addition, intravascular navigation can be performed to determine the extent of blood vessel involvement. The planner includes the ability to describe anatomical variations, both vascular and biliary, offering critical information for planning complex surgical procedures. Additionally, it facilitates the intraluminal navigation of tubular structures, such as blood vessels and bile ducts, providing a unique and precise internal perspective. Moreover, it supplies tools for calculating volumes based on Couinaud’s anatomical segmentation, allowing detailed analysis of hepatic and lesion volumes (video – Online Resource 1).

### Statistical analysis

A descriptive analysis of the study population was conducted, and continuous variables were expressed as mean ± standard deviation (SD) or median with interquartile range (IQR), depending on the data distribution assessed by the Shapiro-Wilk test. Categorical variables were presented as absolute frequencies and percentages. Arterial complications were chosen for the evaluation of 3D model diagnostic accuracy in describing hepatic anatomy because they were the only complications with the reliable intraoperative feedback of the site of anastomosis. A comparison between the thrombosis/stenosis extension described by the virtual model and the actual site of the new arterial anastomosis at retransplantation (gold standard) was performed. The concordance rate of the 3D model was calculated, and overall accuracy and sensitivity were estimated. A one-sided binomial test was performed to determine whether the observed concordance rate was significantly higher than that expected by chance. In addition, a questionnaire (Online Resource 2) was submitted to the five experienced surgeons of the team to assess the perceived benefit of 3D reconstructions through a Likert scale (1 to 5). The internal consistency of the questionnaire was assessed using Cronbach’s α coefficient, with values ≥ 0.70 considered acceptable for reliability. All analyses were performed using SPSS software, version 25 (IBM Corp., Armonk, NY, USA) and statistical significance was defined as *p* < 0.05.

## Results

A total of 39 cases of LT-related complication were included in the study, according to selection criteria: specifically, 17 (43.6%) arterial complications, 11 (28.2%) PVT and 11 (28.2%) biliary strictures. Median age was 58 years (54–63) and 87.2% were male (Online Resource 3).

### Hepatic artery complications

**Fig. 1:**
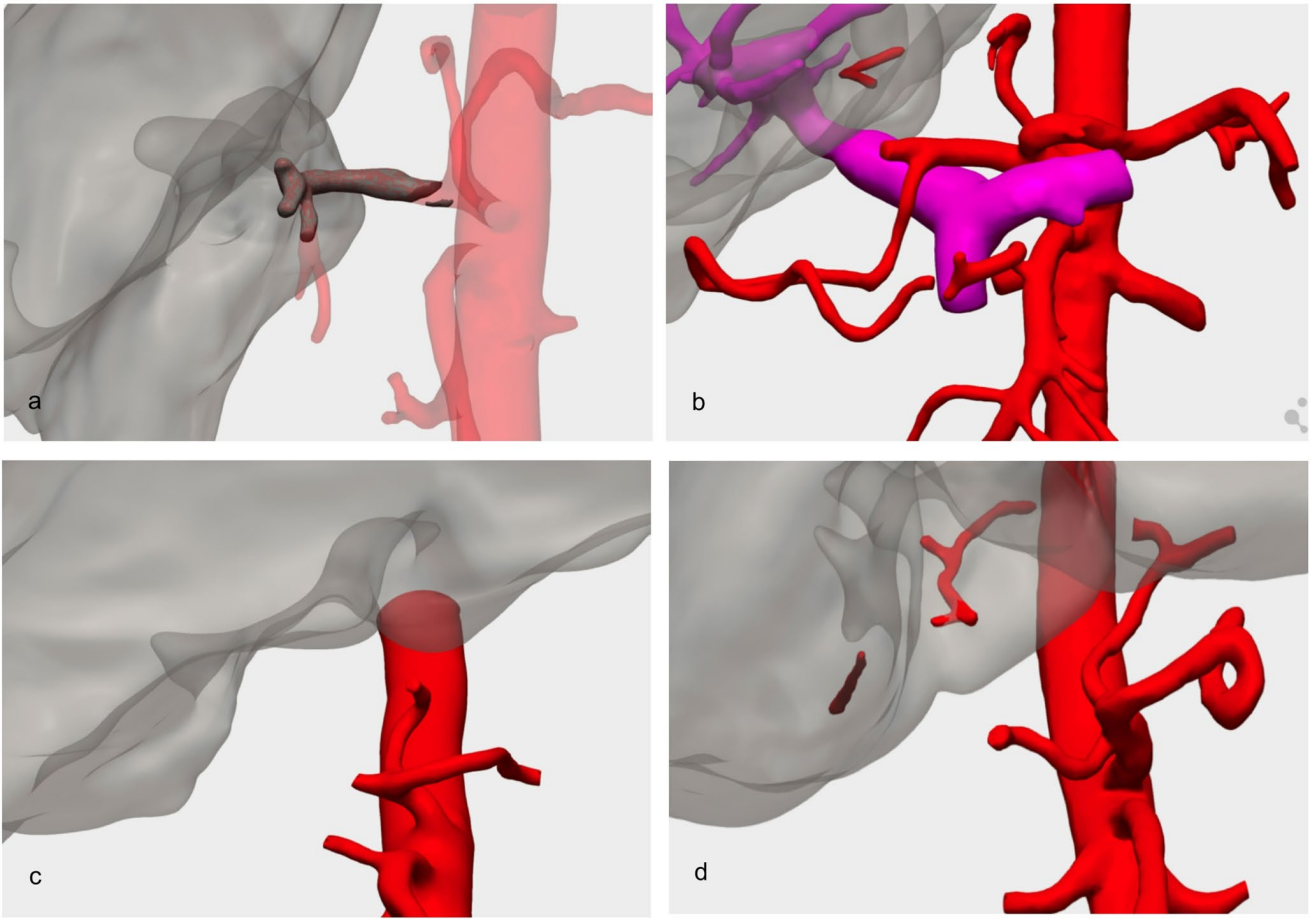
Examples of 3D reconstructions of arterial complications. **a **Hepatic artery thrombosis with virtual reconstruction of a thrombus extending from the origin of the graft-CHA until the bifurcation of the RHA and LHA. **b** Celiac trunk 3D-reconstruction of a CHA stenosis with preserved bifurcation of the PHA-GDA, amputated PHA, and GDA arch to the SMA. **c** Celiac trunk and SMA reconstruction with abrupt occlusion of the CHA and replaced-RHA from the SMA. **d** Amputation of CHA with distal repermeabilization. CHA= Common Hepatic Artery; RHA = Right Hepatic Artery; LHA = Left Hepatic Artery; PHA = Proper Hepatic Artery; GDA = GastroDuodenal Artery; SMA = Superior Mesenteric Artery

Sixteen (94.1%) thrombosis (HAT) and only 1 case of anastomosis’ stenosis were observed (Fig. 1). The median time of occurrence was 5 (1–24.5.5) days, 11 (64.7%) patients developed parenchymal ischemia, extended to 3 or more liver segments in 41.2% of cases, and two patients (11.8%) exhibited ischemic cholangiopathy as a HAT consequence. The prevalent donor type was donation after brain death (DBD; 82.4%) and the most common anastomoses were between donor common hepatic artery and receptor common hepatic artery bifurcation into proper and gastroduodenal artery (*n* = 6, 35.3%) and donor celiac trunk and receptor common hepatic artery bifurcation into proper and gastroduodenal (*n* = 5, 29.4%). All arterial complications were ultimately managed with retransplantation, although in 2 cases an attempt of revascularization by interventional radiology was made. Table 1 describes the arterial anastomosis at the first LT and at retransplantation. The observed concordance rate between 3D models’ reproductions of arterial thrombosis extension and new site of anastomosis at transplantation was 88.2% (15/17), as reported in Table 2. An exact one-sided binomial test confirmed it was significantly higher than expected by chance (*p* = 0.0012), with a 95% one-sided confidence interval indicating a true concordance of at least 67.4%.

**Table 1:** Arterial anastomosis donor-receptor combinations at first liver transplant and at re-transplantation

1 st LT		Re-LT	
Donor-graft	Receptor	Donor-graft	Receptor
SA-CHA	CHA	Celiac trunk	SA-CHA
Celiac trunk	CHA-GDA	Celiac trunk	SA
CHA	SA-CHA	Celiac trunk	SA-CHA
CHA	CHA-GDA	CHA	SA-CHA
Celiac trunk	CHA-GDA	CHA-GDA	CHA-GDA
Celiac trunk	CHA-GDA	Celiac trunk	Aorta (goretex graft)
Celiac trunk	CHA (SMA)	Celiac trunk	CHA (SMA)
Celiac trunk	Aorta (goretex graft)	SMA	Aorta (goretex graft)
CHA	CHA-GDA	CHA	CHA
Celiac trunk	RHA-LHA	Celiac trunk	Celiac trunk
CHA	CHA-GDA	CHA	Celiac trunk
CHA	CHA-GDA	CHA	CHA-GDA
Celiac trunk	CHA-GDA	Celiac trunk	Celiac trunk
CHA	CHA-GDA	Celiac trunk	CHA-GDA
CHA	CHA-GDA	Celiac trunk	Celiac trunk
SMA	CHA-GDA	Celiac trunk	SA
Celiac trunk	RHA-LHA	Celiac trunk	SA

*SA* splenic artery, *CHA* common hepatic artery, *GDA* gastrodudenal artery, *SMA * superior mesenteric artery, *RHA* right hepatic artery, *LHA* left heaptic artery

**Table 2:** Concordance between 3D model thrombosis/stenosis extension and site of new anastomosis at retransplantation

Thrombosis/stenosis location on 3D reconstruction	Site of new anastomosis at retransplantation	Concordance
1 cm from the origin of CHA	CHA-SA bifurcation	Yes
Origin of CHA	SA	Yes
CHA	CHA-SA bifurcation	Yes
CHA-GDA	CHA-SA bifurcation	Yes
PHA	CHA-GDA bifurcation	Yes
2 cm form the origin of CHA	Infra-renal aorta (goretex graft)	No
8.5 mm from the origin of CHA (SMA)	CHA (from SMA)	Yes
Aorta (graft)	Aorta (graft)	Yes
PHA + GDA	CHA	Yes
PHA	Celiac trunk	No
2 cm from the origin of CHA	Celiac trunk	Yes
PHA-GDA	CHA	Yes
1 cm from the origin of CHA	Celiac trunk	Yes
1.2 cm from the origin of PHA	CHA-GDA bifurcation	Yes
1 cm from the origin of CHA	Celiac trunk	Yes
1 cm from the origin of CHA (SMA)	SA	Yes
CHA	SA	Yes
		15/17 (88.2%)

*CHA* common hepatic artery, *SA* splenic artery, *GDA* gastroduodenal artery, *PHA* proper hepatic artery, *SMA* superior mesenteric artery

### Portal vein thromboses

**Fig. 2:**
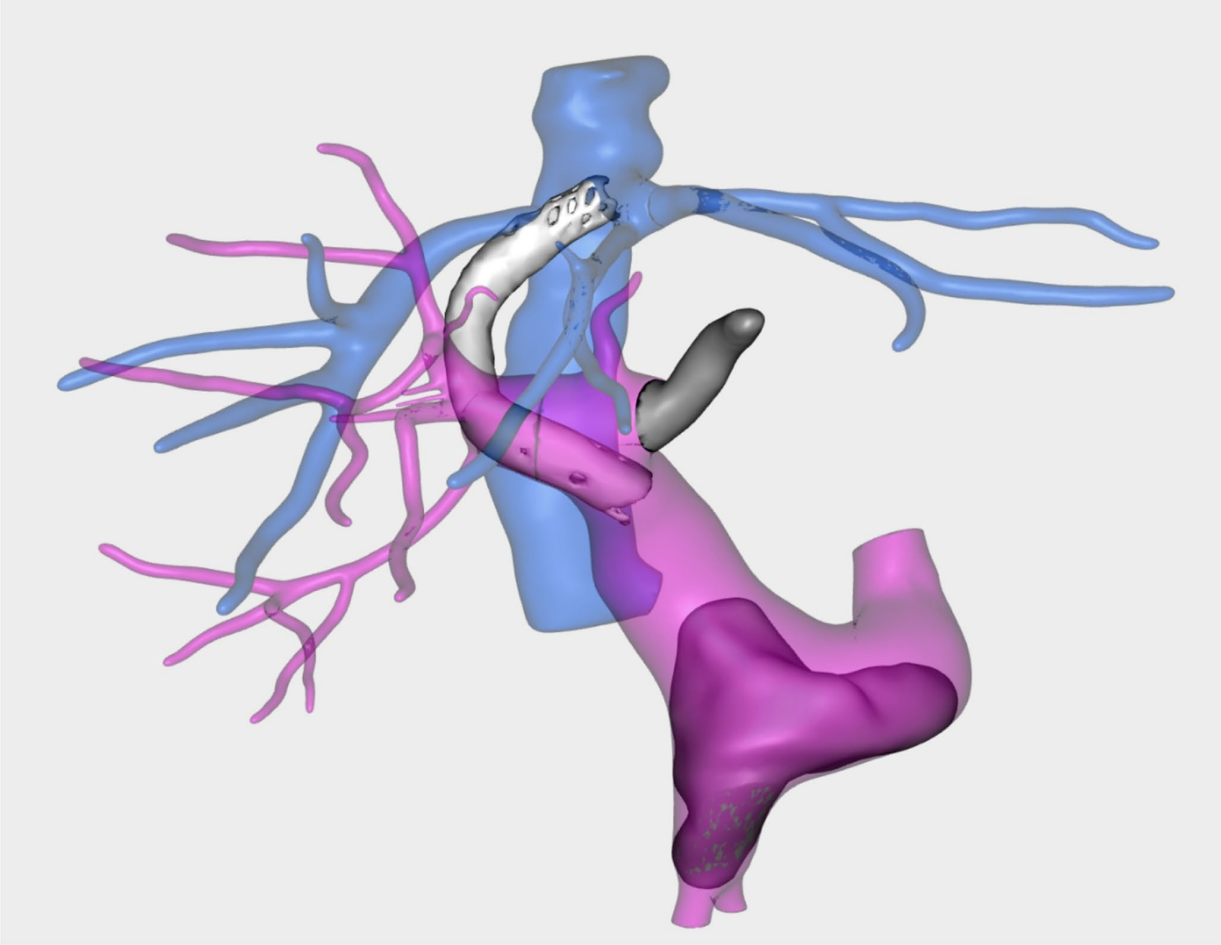
Transjugular Intrahepatic Portosystemic Shunt (TIPS) to treat clinically relevant portal hypertension and subsequent main portal vein thrombosis leading to transplantation. Thrombosis of the spleno-porto-mesenteric confluence (grade IV of Yerdel) and of the main left portal branch. TIPS between the common trunk of the middle and left hepatic veins and distal portal vein

**Fig. 3:**
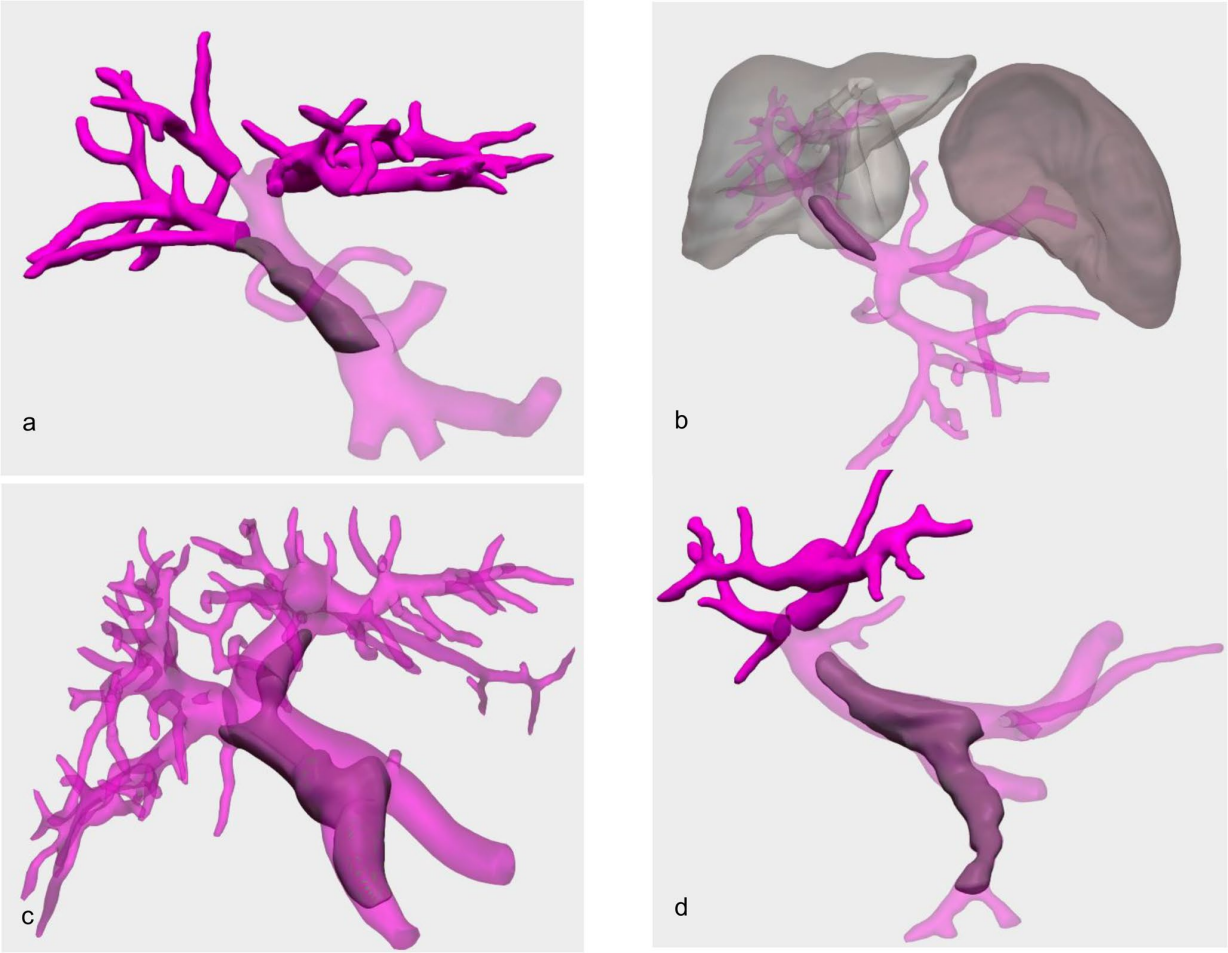
Portal Vein Trombosis (PVT) according to Yerdel grading system. **a** Grade I: partial thrombosis, with the thrombus occupying less than 50% of the portal lumen **b** Grade II: partial thrombosis with the thrombus occupying more than 50% of the portal lumen **c **Grade III: complete portal vein thrombosis with extension into the proximal superior mesenteric vein **d**Grade IV: complete PVT extended to the proximal and distal superior mesenteric vein

Nine patients presented with PVT prior to LT, 1 initially treated with a Transjugular Intrahepatic Porto-systemic Shunt (TIPS – Fig. 2), and 2 in the immediate postoperative course; following the Yerdel grading system, 5 were classified as grade I, 4 as grade II, 1 as grade III and 1 as grade IV (Fig. 3).

DBD was the graft source in 54.5% of cases and an eversion thrombectomy at the time of portal anastomosis was performed in all the cases of preoperative PVT. The 2 postoperative PVT were managed with a prompt retransplantation, which confirmed, in both cases, the site and extension of thrombosis described by the 3D models.

### Biliary strictures

**Fig. 4:**
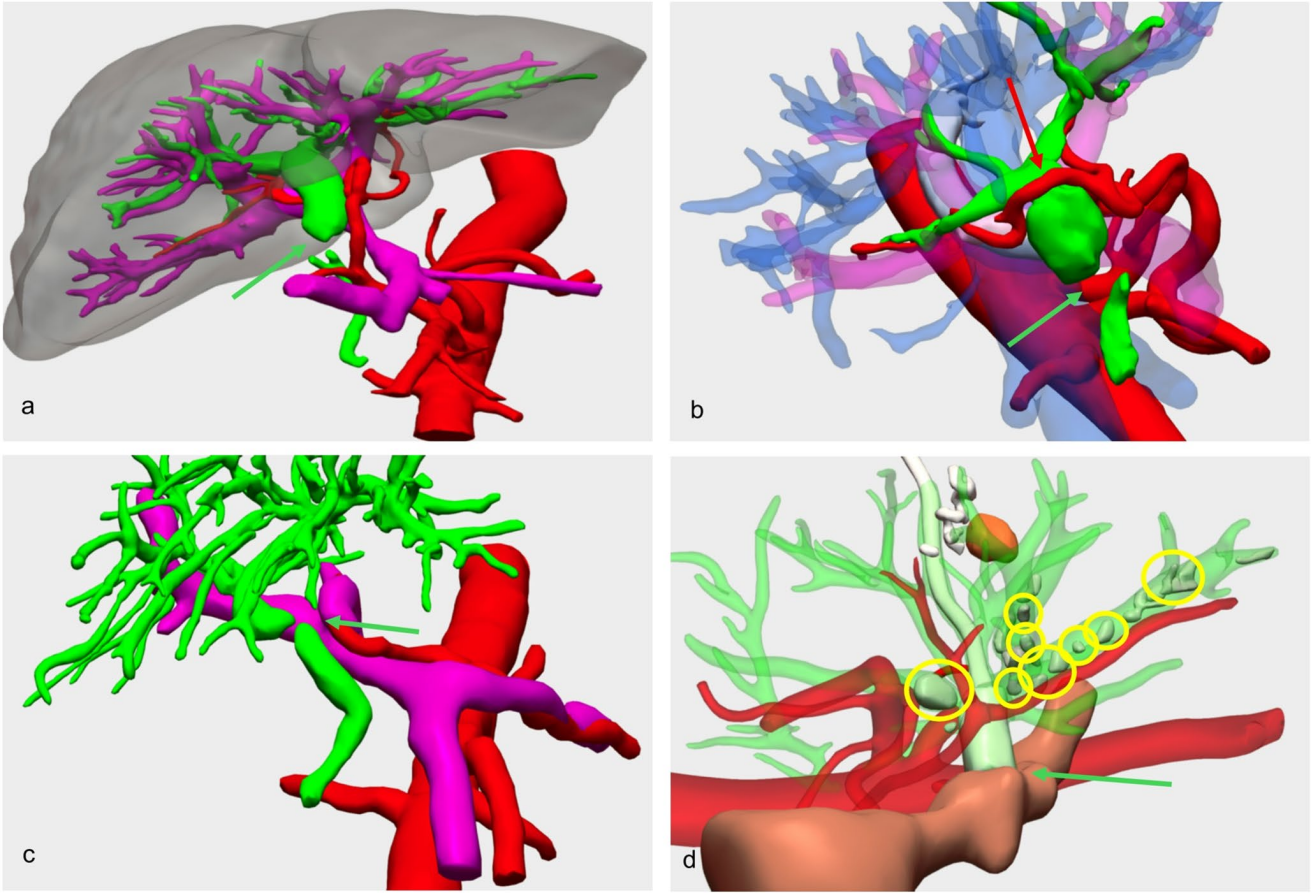
3D reconstructions of biliary stenoses. **a **Biliary stricture of a termino-terminal choledoco-choledocal anastomosis (green arrow). **b** Biliary stricture of a termino-terminal choledoco-choledocal anastomosis (green arrow). Right hepatic artery with preductal pathway as a relevant anatomical variant (red arrow). **c **Proximal stenosis of a termino-terminal choledoco-choledocal anastomosis involving the main biliary duct bifurcation (green arrow). **d **Distal stenosis of a hepatico-jejunostomy (green arrow) associated with intrahepatic lithiasis (yellow circles). Living donor LT with recurrent acute cholangitis. Conservative treatment was attempted first with a percutaneous metal stent, followed by a percutaneous external-internal biliary drainage, but eventually failed and required a new hepatico-jejunostomy

Eleven patients presented with biliary stenoses (Fig. 4), after a median of 5.4 (1.6–29.8) months following LT. The prevalent donor type was, again, DBD (63.6%), with 1 case of living donor LT in a pediatric biliary athresia (Fig. 4D).

Although a conservative treatment was attempted in all cases, either via Percutaneous Trans-hepatic Biliary Drainage (PTBD) or Endoscopic Retrograde Cholangiopancreatography (ERCP), 9 (81.8%) patients eventually required a surgical intervention consisting of 8 hepatic-jejunostomies and one retransplantation for secondary biliary cirrhosis. In all surgically treated patients, the site of the new biliary anastomosis planned on 3D reconstructions agreed with the actual location of its execution.

### Transplant surgeons’ evaluation

Among the transplant surgeons evaluated, the 5 most experienced surgeons of the team (62.5%, with more than 20 years of surgical practice) consistently perceived substantial benefits from the use of 3D reconstructions during preoperative planning, reflected by a mean rating of 4.6/5 and a high internal consistency (Cronbach’s α = 0.936; Table 3).

**Table 3:** Evaluation of surgeons perceived benefit of 3D reconstructions in LT-related complication management

Parameters evaluated	Likert Score		
	Mean (SD)	Median (IQR)	Mode
Understanding of complex arterial anomalies in liver transplant candidates/recipients	4.8 ± 0.45	5 (4.5–5.5)	5
Pre-operative planning of arterial reconstruction	4.4 ± 0.89	5 (3.5–5.5)	5
Improved diagnostic accuracy for portal vein complications before and after liver transplantation	4.2 ± 0.84	4 (3.5–5.5)	4
Aid in the surgical planning for complex portal vein revisions or shunts	4.6 ± 0.55	5 (4–5)	5
Help identifying and characterizing biliary tree complications	4.8 ± 0.45	5 (4.5–5.5)	5
Improved communication inside multidisciplinary team	4.8 ± 0.45	5 (4.5–5.5)	5
Routinely application into the diagnostic and planning workflow for liver transplant complications	4.6 ± 0.89	5 (4–5)	5

## Discussion

This study suggests that the use of cutting-edge 3D modeling allows for a better understanding of vascular and biliary anatomy, both in preoperative planning for LT and in addressing associated complications, which can aid in tailoring treatment to each patient. Three-dimensional reconstructions accurately reproduced the real arterial complication in 88.2% of cases in our series and the expert surgeons of the team found the virtual model assistant very useful. In the perioperative setting, three major challenges arise related to portal, arterial, and biliary complications. The standard surgical approach involves direct portal vein anastomosis, but this may not be feasible in the presence of portal vein thrombosis (PVT), which can obstruct the connection. In such cases, thrombectomy or bypass may be required. Portal vein complications often occur at anastomotic sites, highlighting the importance of precise localization. Patients with grade IV PVT (Yerdel) have higher postoperative mortality, with a one-year mortality rate of 42% in complete PVT cases, compared to 22% in partial PVT [15]. Previously, PVT was a contraindication for LT in cirrhotic patients, but advances in diagnostics, medical therapy, and surgical techniques have made transplantation possible even in these cases [16]. Preoperative imaging is essential before listing patients for LT to plan the best approach if standard porto-portal anastomosis is not feasible. 3D modeling provides a detailed visualization of thrombosis severity, thrombus extension, TIPS presence and vascular relationships, improving preoperative appraisal. In our experience, virtual models have always correctly assessed the PVT grade and the precise anatomy of the spleno-porto-mesenteric confluence.

Arterial anastomosis complications following LT are infrequent but represent some of the most severe issues, resulting in high rates of graft loss and even mortality [17, 18]. Moreover, they can affect biliary anastomoses, leading to biliary strictures, chronic cholangitis, biliary abscesses, sepsis, and death by compromising the vascular source of the biliary tree [12]. In our study, 3D models were particularly useful in preoperative retransplantation planning, providing a reliable representation of the caliber, trajectory and spatial relationships of the celiac trunk and aortic branches, the type of anatomical variants, the arterial stenosis site and the presence of atherosclerotic calcification not clearly visualized by axial cuts of conventional radiology. Through such preoperative insight, surgeons can anticipate technical difficulties and determine whether additional vascular grafts or endarterectomy procedures may be required, resulting in shorter ischemia time and improved graft perfusion, ultimately leading to better transplant outcomes [19]. The concordance rate between 3D arterial reconstruction and intraoperative findings was high (88.2%), with only two cases of discrepancy, represented by two complex post-transplant anatomy, one with a pre-existent aortic graft. These cases highlight the inherent challenges in vascular complication assessment after LT and emphasize the need for 3D models caution interpretation within a multidisciplinary network.

Biliary complications affect up to 25% of transplant recipients [19], and their incidence varies depending on the donor type. Biliary strictures (40% of all biliary complications) represent the most prevalent post-LT complications [20, 21]. Primary causes of biliary strictures include the type of liver transplant procedure, surgical techniques employed, T-tube usage, hepatic artery thrombosis development, and the occurrence of ischemic lesions within the biliary ducts [20, 22]. Diffuse and multifocal strictures are often attributed to the latter group of factors. The integration of advanced 3D imaging technology plays a pivotal role in the diagnosis and management of biliary complications, allowing the precise characterization of biliary strictures, identification of bile duct stones (choledocholithiasis) and ischemic cholangiopathy. The location, length, and severity of biliary strictures, whether anastomotic or not, are crucial for treatment selection (e.g. endoscopic stenting, percutaneous dilation or surgical revision with hepaticojejunostomy). Additionally, in the preoperative setting, virtual 3D reconstruction facilitates aberrant arterial pathway recognition, such as preductal ones, as well as the presence, exact number, size, and anatomical site of bile duct stones, aiding in choosing the least invasive and most effective intervention. In ischemic cholangiopathy, 3D imaging facilitates detailed visualization of intrahepatic bile ducts, helping to distinguish isolated strictures from diffuse damage or complete ductal necrosis. This accuracy contributes to better patient selection and more tailored treatments, reducing the risk of incomplete intervention and preventing early retransplantation.

From the questionnaire distributed to our expert team, it was clear that 3D reconstructions are perceived as highly beneficial not only for individual surgical planning, but also for supporting multidisciplinary decision-making (4.8/5) [23]. In complex clinical scenarios, such as LT, the visual clarity provided by 3D models can help harmonize perspectives between surgeons, radiologists, anesthesiologists, and hepatologists, thus fostering more informed and shared clinical decisions. For the same reason, 3D models have already demonstrated relevant didactic value, particularly for training junior surgeons or explaining anatomy to medical students and residents [23–25]. 

Next-generation 3D modeling has already found wide application in various surgical fields and within the realm of hepatobiliary surgery, is has already been employed to evaluate tumor relationships with vascular and biliary anatomy, thereby guiding segmental or lobar resections [26, 27]. Its application is particularly advantageous in discerning intricate small vessel anatomy, as conventional 2D imaging methods alone often fail to accurately depict the branching patterns of hepatic arteries [3]. However, despite these advancements, its’ implementation in LT remains limited. This is partly due to the complexity of transplant logistics and partly due to the lack of standardization in 3D reconstruction workflows tailored to transplant-specific challenges.

Although not represented in our cohort, the subgroup of living donor liver transplantation (LDLT) patients has thus far shown the most compelling results regarding the clinical application of 3D modeling. Within the domain of LDLT, 3D reconstruction techniques have emerged as invaluable tools for accurate and efficient evaluation of liver vascular systems, liver dimensions, and volumes in both donor and recipient organs. Simulation surgery facilitated by 3D reconstruction enables enhanced decision-making regarding resection levels, vascular management, and donor liver reconstruction [3, 28–30]. These benefits are particularly relevant in a field where surgical precision and donor safety are of paramount importance, and where preoperative visualization can directly impact both technical feasibility and outcomes. Furthermore, virtual hepatectomy, made possible through these techniques, contributes to transplant success by optimizing donor selection, venous reconstruction decisions, and equilibrium between recipient and donor situations [31]. Additionally, the assessment of the bile duct system using MRI adds significant value to the process of living donor liver transplantation [32].

The potential integration of 3D models into routine clinical practice deserves consideration. Once the workflow is standardized, 3D reconstructions can be generated using imaging studies already acquired as part of standard clinical care (CT or MRI), without the need for additional examinations. The processing time required for each patient appears compatible with clinical decision-making, particularly in complex post–liver transplantation scenarios where vascular or biliary complications are suspected. Nevertheless, the implementation of this approach relies on the availability of trained personnel for image segmentation and reconstruction, as well as access to dedicated software. These resources are increasingly present in tertiary referral and transplant centers. Within this framework, the 3D model may be best suited for selected, complex cases in which it can offer incremental value for diagnostic assessment and therapeutic planning, rather than being adopted as a routine tool for all transplant recipients.

This study has limitations that must be acknowledged. First, as a retrospective pilot study, it is inherently subject to selection and information biases related to its design. The small sample size and lack of prospective validation limit the generalizability of our findings. Additionally, intraoperative data, which were used as the gold standard for assessing the diagnostic accuracy of 3D reconstructions on arterial complications, were nearly always incomplete or missing regarding degree of portal thrombosis and extent of biliary strictures, thus making an assessment of concordance for these complications impossible. Despite these limitations, we believe that this study provides important preliminary evidence supporting the expansion of next-generation 3D modeling applications in the field of LT. More extensive analysis, including data on PVT and biliary complications as well as standardized protocols for integrating 3D modeling into routine transplantation workflows are needed, to ensure its optimal use and improve surgical safety and efficacy.

## Conclusions

This study suggests that next-generation 3D modeling could provide significant advantages in the preoperative and intraoperative assessment of LT, particularly for managing arterial, portal, and biliary complications. The ability to create detailed patient-specific anatomical reconstructions enhances surgical planning, allowing for more precise decision-making and potentially reducing the incidence of severe postoperative complications. While further large-scale studies are warranted to validate these findings, 3D imaging technology appears to be a valuable tool in LT, enhancing precision medicine and facilitating communication and training within multidisciplinary teams.

## Supplementary Information

Below is the link to the electronic supplementary material.

Supplementary Material 1 (MP4 95.2 MB) 

Supplementary Material 2 (PDF 105 KB)

Supplementary Material 3 (DOCX 21.0 KB)

Supplementary Material 4 (DOCX 35.2 KB)

## Data Availability

No datasets were generated or analysed during the current study.

## Declarations

### Reporting checklist

The authors have completed the STROBE reporting checklist (online resource 4).
