# Supplementary material for: Personalized 3D surgical planning in liver transplantation: A new era in preoperative assessment and management of vascular and biliary complications
Source: Langenbecks Arch Surg. 2026 Feb 27;411(1):105. doi: 10.1007/s00423-026-03984-w (PMC13002731; doi:10.1007/s00423-026-03984-w)
Supplement: Supplementary file 2 — Supplementary Material 2 (PDF 105 KB) [file 423_2026_3984_MOESM2_ESM.pdf]

Evaluation of 3D Model Utility in Liver Transplant Complications

Thank you for participating in this survey. Your responses are crucial for understanding the perceived utility of 3D anatomical models in the management of portal vein thrombosis, arterial thrombosis and biliary stenosis after liver transplantation. This questionnaire is completely anonymous, and all responses will be used solely for aggregated statistical purposes. No personal data will be collected or associated with your answers.

* Indicates required question

1. How many years of experience do you have in liver transplantation or hepatobiliary surgery? *

Mark only one oval.

- 0-5 years
- 5-10 years
- 10-20 years
- more than 20 years

2. 3D models enhance the understanding of complex arterial anomalies in liver transplant candidates/recipients *

Mark only one oval.

	1	2	3	4	5	
stro	◯	◯	◯	◯	◯	strongly agree

3. 3D models are useful for pre-operative planning of arterial reconstruction in cases of complications (e.g., hepatic artery thrombosis, stenosis) *

Mark only one oval.

	1	2	3	4	5	
stro	◯	◯	◯	◯	◯	strongly agree

4. The use of 3D models improves diagnostic accuracy for portal vein complications (e.g., extension of thrombosis, stenosis) before and after liver transplantation. *

Mark only one oval.

	1	2	3	4	5	
stro	◯	◯	◯	◯	◯	strongly agree

5. 3D models aid in the surgical planning for complex portal vein revisions or shunts *

Mark only one oval.

	1	2	3	4	5	
stro	◯	◯	◯	◯	◯	strongly agree

6. 3D models provide valuable insights for identifying and characterizing biliary tree complications (e.g., strictures, leaks) after liver transplantation *

Mark only one oval.

	1	2	3	4	5	
stro	◯	◯	◯	◯	◯	strongly agree

7. Overall, 3D models improve communication among the multidisciplinary team (surgeons, radiologists, hepatologists) when discussing complex cases *

Mark only one oval.

	1	2	3	4	5	
stro	◯	◯	◯	◯	◯	strongly agree

8. I believe 3D models should be routinely incorporated into the diagnostic and planning workflow for liver transplant complications *

Mark only one oval.

	1	2	3	4	5	
stro	◯	◯	◯	◯	◯	strongly agree

This content is neither created nor endorsed by Google.

Google Forms
