# Supplementary material for: Personalized 3D surgical planning in liver transplantation: A new era in preoperative assessment and management of vascular and biliary complications
Source: Langenbecks Arch Surg. 2026 Feb 27;411(1):105. doi: 10.1007/s00423-026-03984-w (PMC13002731; doi:10.1007/s00423-026-03984-w)
Supplement: Supplementary file 3 — Supplementary Material 3 (DOCX 21.0 KB) [file 423_2026_3984_MOESM3_ESM.docx]

**Online resource 3**

**Table S1A. Summary of arterial complications in liver transplant recipients**

| **Variables** | **N=17** |
| --- | --- |
| Age (years) | 57 (53-61) |
| Sex (male) | 15 (88.2%) |
| Indication of liver transplantation   - Alcohol abuse - Viral hepatitis (HBV, HCV) - Hepatocarcinoma over chirrosis - Acute liver failure - Others† | 3 (17.6%)  1 (5.9%)  6 (35.3%)  2 (11.8%)  5 (29.4%) |
| Donor type   - DBD - DCD | 14 (82.4%)  3 (17.6%) |
| Type of arterial complication   - Thrombosis - Stenosis | 16 (94.1%)  1 (5.9%) |
| Time to diagnosis (days) | 5 (1-24.5) |
| Associated complications   - Parenchymal ischemia - ≥ 3 segments - Ischemic cholangitis | 11 (64.7%)  7 (41.2%)  2 (11.8%) |
| Arterial complication management:   - Re-LT | 17 (100%) |
| Donor type at re-LT   - DBD - DCD | 16 (94.1%)  1 (5.9%) |

†1 autoimmune hepatitis, 1 metastases of ileal neuroendocrine tumor, 1 Budd-Chiari, 1 biliary stenosis of previous LT, 1 chronic rejection of previous LT

DBD = death-brain donor; DCD = death-cardiac donor; HBV = hepatitis B virus; HCV = hepatitis C virus

**Table S1B. Summary of portal vein thrombosis in liver transplant recipients**

| **Variables** | **N=11** |
| --- | --- |
| Age | 63 (58-65) |
| Sex (male) | 8 (72.7%) |
| Indication of liver transplantation   - Alcohol abuse - Viral hepatitis (HBV, HCV) - Hepatocarcinoma over cirrhosis - Acute liver failure - Others† | 4 (36.4%)  2 (18.2%)  2 (18.2%)  0 (0%)  3 (27.3%) |
| Donor type   - DBD - DCD | 6 (54.5%)  5 (45.5%) |
| TIPS | 1 (9.1%) |
| Timing of PVT   - Pre-LT - Post-LT | 9 (81.8%)  2 (18.2%) |
| PVT grade (Yerdel)   - Grade I - Grade II - Grade III - Grade IV | 5 (45.5%)  4 (36.4%)  1 (9.1%)  1 (9.1%) |
| PVT management‡:   - Eversion thrombectomy - Re-LT | 9 (81.8%)  2 (18.2%) |

**†**1 cryptogenic cirrhosis; 1 primary biliary cholangitis; 1 ischemic cholangitis following previous LT

‡Correspond to PVT timing: all pre-LT PVT were treated with eversion thrombectomy; all post-LT PVT required re-transplantation

HBV = Hepatitis B Virus; HCV = Hepatitis C Virus; DBD = death-brain donor; DCD = death-cardiac donor; TIPS = Transjugular Intrahepatic Portosystemic Shunt; PVT = portal vein thrombosis.

**Table S1C. Summary of biliary complications in liver transplant recipients**

| **Variables** | **N=11** |
| --- | --- |
| Age | 56 (43-60) |
| Sex (male) | 11 (100%) |
| Indication of liver transplantation   - Alcohol abuse - Viral hepatitis (HBV, HCV) - Hepatocarcinoma over chirrosis - Acute liver failure - Others† | 2 (18.2%)  2 (18.2%)  5 (45.5%)  0  2 (18.2%) |
| Donor type   - DBD - DCD - Living donor | 7 (63.6%)  3 (27.3%)  1 (9.1%) |
| Type of biliary complication   - Biliary stenosis - Choledocolitiasis / intrahepatic litiasis - Recurrent cholangitis | 6 (54.5%)  4 (36.4%)  1 (9.1%) |
| Time to diagnosis (months) | 5.4 (1.6-29.8) |
| Type of biliary anastomosis   - Choledoco-choledocal without T-tube - Choledoco-choledocal with T-tube - Hepaticojejunostomy | 8 (72.7%)  2 (18.2%)  1 (9.1%) |
| Biliary complication management:   - ERCP - Hepaticojejunostomy - Re-transplantation | 2 (18.2%)  8 (72.7%)  1 (9.1%) |

†1 cryptogenic cirrhosis; 1 biliary atresia

HBV = Hepatitis B Virus; HCV = Hepatitis C Virus; DBD = death-brain donor; DCD = death-cardiac donor; ERCP = endoscopic retrograde cholangiopancreatography
